# Hommage à Madame le Professeur Maryvonne Kombila (1946-2024)

**DOI:** 10.48327/mtsi.v4i4.2024.580

**Published:** 2024-10-24

**Authors:** Dominique RICHARD-LENOBLE

**Affiliations:** Clos Sainte Roselle, 6 bis rue Saint Venant, 37230 Luynes, France

## Mbolo !

**Figure 1:**
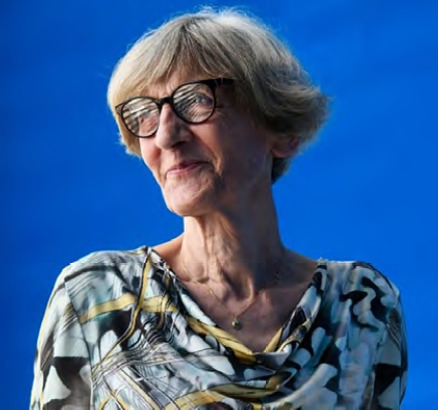
Pr. Maryvonne Kombila (crédit photo : archive familiale)

Née en 1946 d'humbles paysans métayers dans les pays de Loire, Maryvonne Favry, brillante et rebelle, d'après son père, est orientée à l’âge de 10 ans vers un pensionnat catholique où elle fait de remarquables études. Elle obtient un baccalauréat en philosophie qui ne parait pas suffisant à ses parents. Ils la trouvent trop jeune et lui feront soutenir l'année suivante un baccalauréat scientifique.

Elle entre à la faculté de médecine de Rennes où elle est voisine d'amphithéâtre de Pierre André Kombila. Tous les deux, très actifs dans les mouvements étudiants, forment déjà un couple fusionnel, passionnel, battant, luttant contre les communautarismes, les maltraités de toutes origines. Ils prendront dans les associations étudiantes et pour toujours, la défense des plus faibles. Tandis que Pierre, qu'elle vient d’épouser, suit un brillant *curriculum* en cardiologie, elle s'oriente rapidement vers la médecine tropicale, la parasitologie et la mycologie. Sa formation en biologie médicale se poursuit à l’Institut Pasteur. Maryvonne soutient sa thèse en 1973. Tous les deux assistants puis maîtres de conférences, ils gravissent les échelons qui les conduisent au titre de professeur. En cardiologie pour Pierre, qui sera pour cette discipline le premier professeur agrégé au service du Gabon. Maryvonne sera pour sa part la première professeure agrégée en parasitologie-mycologie-médecine tropicale de ce qui était à l’époque le Centre universitaire des Sciences de la santé gabonais (CUSS) qu'ils ont intégré en 1978.

Ils décident en 1978-79 de quitter leurs fonctions hospitalo-universitaires françaises pour intégrer le Centre hospitalo-universitaire des sciences de la santé (CUSS) à Libreville.

Dans un service d'enseignement de soins et de recherche en Santé internationale en construction, j'ai le grand honneur d'accueillir Madame le Professeur Maryvonne Kombila comme collaboratrice. Nous partageons au CUSS nos fonctions universitaires, de soins et de recherche. Sa compétence, son opiniâtreté au travail, et son honnêteté, que ses amis français lui connaissaient, sont immédiatement reconnues par nos confrères gabonais et, plus largement, d’Afrique équatoriale. Sans concession, avec acharnement, elle défend ses idées et les discussions autour d'un café dans notre service sont fréquentes, enrichissantes, passionnées. Jacques Chandenier, Frederick Gay, Dominique Gendrel, André Moussavou, Margarita Gomez de Diaz, Jean-Luc Moreno, Marie-Louise Maganga, Muriel Nicolas, Mady Thérizol, Olivier Mariotte, et bien d'autres, viennent débattre et partager leurs expériences. Adorée, respectée des étudiants pour sa rigueur et son sérieux, elle sait transmettre les expériences de terrain acquises au cours de missions menées dans les villages gabonais sur l'ensemble du territoire. Elle enseigne à tous les niveaux et crée un institut de formation pour les biotechnologistes au sein de l'université (TSBM).

Elle inaugure et assure l’éclat d'une consultation de dermato-mycologie, unique en Afrique centrale. Elle vit plus proche des malades et des maladies que des thermocycleurs, pourtant indispensables et en bonne place dans son laboratoire de recherche. Elle possédait toutes les qualités nécessaires à une recherche de terrain : énergie physique, patience, opiniâtreté, compétence. Nous partagions des missions passionnantes où presque tout restait à découvrir, dans le domaine des filarioses, avec Jacques Chandenier pour l'onchocercose, ou encore avec Dominique Gendrel et Éric Pichard pour les aspects cliniques des distributions communautaires des programmes Mectizan™ testés et développés au Gabon. Nous avons découvert à une heure du matin, près de Yombi, une microfilaire de *Mansonella rodhaini* pour la première fois chez l’Homme de même que *Bertiella studeri,* tænia décrit au Gabon en collaboration avec notre assistante Marie-Louise Maganga et Nicole Léger. Le paludisme a permis à Maryvonne de former ses élèves, les Professeurs Marielle Bouyou, Solange Nzenze et Jean-Bernard Lekana, purs produits de son exemple et défenseurs de la compétence et de la rigueur scientifique indispensables à l’Université Gabonaise. Comme eux, tous les jeunes médecins, scientifiques ou chercheurs qui nous accompagnaient dans les missions dites « de brousse », sont restés marqués par sa vitalité, sa passion, son efficacité.

Le scientisme n’était pas son fort. C’était une scientifique équilibrée au service de tous les malades dans leur environnement. Elle développait des recherches cliniques, diagnostiques et thérapeutiques appliquées aux dermato-mycoses africaines, à la trypanosomiase, aux bilharzioses dont elle a partagé l'intérêt avec Thanh Hai Duong et Krystina Mengue Me Ngou Milama dans la description d'une espèce hybride naturelle de schistosome. Sa reconnaissance nationale, continentale et internationale lui permettait d’établir des liens étroits avec d'autres chercheurs comme Albert Samé Ekobo, notre collègue camerounais, Francis Louis, mais aussi notre regrettée Odile Bain du Muséum national d’Histoire naturelle de Paris.

Maryvonne aura été une femme de convictions, rigoureuse et courageuse. En France, dans les moments difficiles, elle sera inconditionnellement soutenue par ses frères de l'ouest, les Professeurs Claude Guiguen, Dominique Chabasse mais aussi Michel Miegeville et d'autres. Au Gabon, elle défend ses idées comme celles de son mari et, malgré de lâches attaques, elle maintiendra le cap et gardera la tête haute. Pas toujours en accord avec les décisions politiques de son pays, le Gabon, elle parlait haut, laissait faire et laissait dire sans faiblir, sans concession dans le discours. Elle était un soutien sans faille pour son mari. Elle formait avec Pierre un couple au métissage exemplaire au milieu d'un communautarisme destructeur toujours ambiant.

Comme le rappelait mon amie Dorothée Kindé-Gazard, présidente de la Société africaine de parasitologie et mycologie (SoAP), lors des obsèques de Maryvonne, « Madame Kombila, dans sa vie professionnelle comme familiale, était à l’écoute de tous, intègre, toujours au travail, disponible, avec passion, rigueur, humilité, discrétion, compétence. Courageuse, elle était entière, intransigeante, d'une efficacité pragmatique, au plus proche des problèmes de santé des Gabonais, sans considération de clan, d'ethnie ou de fortune ».

Son expertise et son engagement, ont participé à la formation de nombreux professionnels de santé au Gabon qui suivent son exemple.

Bravo et merci Maryvonne pour tout ce que tu as fait et ce que tu laisses.

*Dyene nkaza g'orèma.*
