# Supplementary material for: Tribute to Professor Maryvonne Kombila (1946-2024)
Source: Med Trop Sante Int. 2024 Oct 24;4(4):mtsi.v4i4.2024.580. [Article in French] doi: 10.48327/mtsi.v4i4.2024.580 (PMC11892393; doi:10.48327/mtsi.v4i4.2024.580)
Supplement: Related texts

# Research article: Tribute to Professor Maryvonne Kombila (1946-2024)

## Mbolo!

**Figure 1:** Prof. Maryvonne Kombila (photo credit: family archive)

Born in 1946 to modest tenant farmers in the Loire region, Maryvonne FAVRY, brilliant and rebellious according to her father, was sent at the age of 10 to a Catholic boarding school, where she did remarkably well. She earned a bachelor's degree in philosophy, but her parents didn't think that was enough. They thought she was too young, so she took a bachelor of science the following year. She entered the Faculty of Medicine in Rennes, where she was sitting next to Pierre André Kombila in the amphitheater. The two of them were very active in the student movements and already formed a close, passionate couple, fighting against communitarianism and the mistreated of all origins. In their student associations, they would always defend the weakest. While Pierre, whom she had just married, pursued a brilliant curriculum in cardiology, she quickly turned to tropical medicine, parasitology and mycology. She continued her training in medical biology at the Institut Pasteur. Maryvonne defended her doctoral thesis in 1973. Both became assistant professors and then senior lecturers, working their way up to the rank of professor. In cardiology for Pierre, who was the first associate professor in this discipline in Gabon. Maryvonne became the first associate professor of parasitology-mycology-tropical medicine at the then Centre Universitaire des Sciences de la Santé Gabonais (CUSS), which they joined in 1978.

In 1978-79, they decided to leave their university hospital duties in France to join the Centre hospitalo-universitaire des sciences de la santé (CUSS) in Libreville.

I had the great honor of welcoming Professor Maryvonne Kombila as my collaborator in a Department of Teaching, Care and Research in International Health that was still under construction. At CUSS, we shared our academic, nursing and research functions. Her competence, hard work and honesty, well known to her French friends, were immediately recognized by our colleagues in Gabon and more broadly in Equatorial Africa. Uncompromising and relentless, she defended her ideas, and discussions over coffee in our department are frequent, enriching and passionate. Jacques Chandenier, Frederick Gay, Dominique Gendrel, André Moussavou, Margarita Gomez de Diaz, Jean-Luc Moreno, Marie-Louise Maganga, Muriel Nicolas, Mady Thérizol, Olivier Mariotte and many others come to discuss and share their experiences.

Loved and respected by her students for her thoroughness and seriousness, she knew how to pass on the field experience she had gained during her missions in Gabonese villages throughout the country. She has taught at all levels and created a training institute for biotechnologists within the university (TSBM).

She inaugurated a dermato-mycology clinic, the only one of its kind in Central Africa, and made it shine. She lived closer to the patients and the diseases than to the thermocyclers, although they were indispensable in her research laboratory. She had all the qualities needed for field research: physical energy, patience, perseverance and competence. We shared exciting missions where almost everything remained to be discovered, in the field of filariasis, with Jacques Chandenier for onchocerciasis, or with Dominique Gendrel and Éric Pichard for the clinical aspects of community distribution of the Mectizan™ programs tested and developed in Gabon. At one o'clock in the morning, near Yombi, we discovered a *Mansonella rodhaini* microfilaria for the first time in humans, as well as *Bertiella studeri,* a taenia described in Gabon in collaboration with our assistant Marie-Louise Maganga and Nicole Léger. Malaria allowed Maryvonne to train her students, Professors Marielle Bouyou, Solange Nzenze and Jean-Bernard Lekana, pure products of her example and defenders of the competence and scientific rigor indispensable to the Gabonese University. Like them, all the young doctors, scientists and researchers who accompanied us on our “bush” missions were marked by his vitality, passion and efficiency.

Scientism was not her strong point. She was a balanced scientist at the service of all patients in her environment. She developed clinical, diagnostic and therapeutic research on African dermatomycoses, trypanosomiasis and bilharziasis and shared an interest with Thanh Hai Duong and Krystina Mengue Me Ngou Milama in the description of a natural hybrid species of schistosome. Her national, continental and international recognition allowed her to establish close links with other researchers such as Albert Samé Ekobo, our Cameroonian colleague Francis Louis and the late Odile Bain of the Muséum national d’Histoire naturelle in Paris.

Maryvonne was a woman of conviction, thoroughness and courage. In France, in difficult times, she was unconditionally supported by her brothers from the West, Professors Claude Guiguen, Dominique Chabasse, Michel Miegeville and others. In Gabon, she defended her ideas and those of her husband, and despite cowardly attacks, she stayed the course and held her head high. Not always in agreement with the political decisions of her country, Gabon, she spoke out, let it happen and let it be said, without wavering, without making concessions in her discourse. She was a tireless supporter of her husband. She and Pierre were an exemplary interracial couple in the midst of the destructive communitarianism that still prevails.

As my friend Dorothée Kindé-Gazard, President of the Société africaine de parasitologie et mycologie (SoAP), recalled at Maryvonne's funeral, “Madame Kombila was attentive to everyone, both in her professional life as well as in her family life, honest, always hardworking, available, with passion, thoroughness, humility, discretion and competence. She was courageous, wholehearted, uncompromising, pragmatically efficient, close to the health problems of the Gabonese people, regardless of clan, ethnic group or wealth”.

Her expertise and dedication have contributed to the training of many health professionals in Gabon who are following her example.

Bravo and thank you Maryvonne for all you have done and all you are leaving behind.

*Dyene nkaza gorèma.*
